# Chronic or Changeable Infarct Size after Spontaneous Coronary Artery Dissection

**DOI:** 10.3390/diagnostics13091518

**Published:** 2023-04-23

**Authors:** Gordana Krljanac, Svetlana Apostolovic, Zlatko Mehmedbegovic, Olga Nedeljkovic-Arsenovic, Ruzica Maksimovic, Ivan Ilic, Aleksandra Djokovic, Lidija Savic, Ratko Lasica, Milika Asanin

**Affiliations:** 1 University Clinical Center of Serbia, Cariology Clinic, Faculty of Medicine, University of Belgrade, 11000 Belgrade, Serbia; 2 Clinical Center of Nis, Cardiology Clinic, Faculty of Medicine, University of Nis, 18000 Niš, Serbia; 3 University Clinical Center of Serbia, Center for Radiology and Magnetic Resonance Imaging, Faculty of Medicine, University of Belgrade, 11000 Belgrade, Serbia; 4 Institute of Cardiovascular Diseases “Dedinje”, Faculty of Medicine, University of Belgrade, 11000 Belgrade, Serbia; 5 University Hospital Center “Bezanijska Kosa”, Department of Cardiology, Division of Interventional Cardiology, Faculty of Medicine, University of Belgrade, 11000 Belgrade, Serbia

**Keywords:** spontaneous coronary artery dissection, cardiac magnetic resonance, strain echocardiography, myocardial injury

## Abstract

Spontaneous coronary artery dissection (SCAD) could be the cause of acute myocardial infarction (AMI) and sudden cardiac death. Clinical presentations can vary considerably, but the most common is the elevation of cardiac biomarkers associated with chest discomfort. Different pathological etiology in comparison with Type 1 AMI is the underlying infarct size in this population. A 42-year-old previously healthy woman presented with SCAD. Detailed diagnostical processing and treatment which were performed could not prevent myocardial injury. The catheterization laboratory was the initial place for the establishment of a diagnosis and proper management. The management process can be very fast and sometimes additional imaging methods are necessary. Finding predictors of SCAD recurrence is challenging, as well as predictors of the resulting infarct scar size. Patients with recurrent clinical symptoms of chest pain, ST elevation, and complication represent a special group of interest. Therapeutic approaches for SCAD range from the ”watch and wait” method to complete revascularization with the implantation of one or more stents or aortocoronary bypass grafting. The infarct size could be balanced through the correct therapeutical approach, and, proper multimodality imaging would be helpful in the assessment of infarct size.

**Figure 1:**
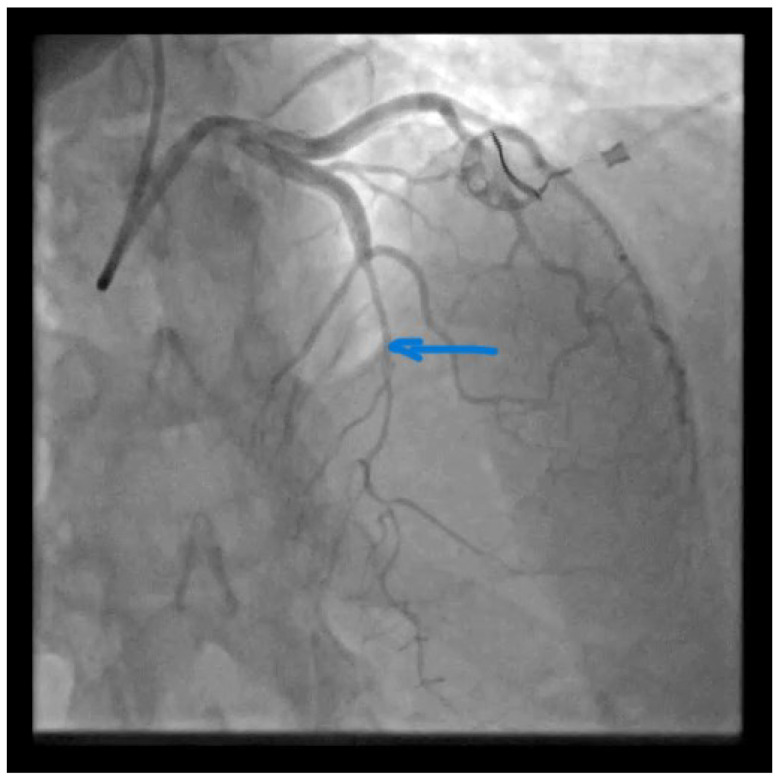
Catheterization of the coronary artery showed spontaneous type 2B coronary artery dissection (SCAD) with thrombolysis in myocardial infarction (TIMI) with flow grade 2 of the left anterior descendent (LAD) coronary artery located in the medial distal segments (blue arrow) where stenosis extends to the end of the vessel (Video S1). SCAD is defined as a nonatherosclerotic or iatrogenic separation of the coronary arterial tunics with a non-traumatic cause, secondary to vasa vasorum hemorrhage or intimal tear, which creates a false lumen, coronary compression, and downstream myocardial ischemia [1]. The primarily affected demographic groups are young-to-middle-aged women [1]. This can sometimes be an unrecognized condition. SCAD can cause acute myocardial infarction (AMI) and sudden cardiac death [2,3]. A previously healthy 42-year-old woman presented to the emergency department with a complaint of gradually squeezing chest pain in rest which lasted for 2.5 h. She had also noticed vomitus and an increased blood pressure value of of 170/120 mmHg. She was admitted to the Cardiology Department at the University Clinical Center of Serbia. No previous diseases were found in her medical history. She underwent five normal deliveries and one spontaneous abortion. The last delivery was eight years ago. She did not use any hormonal therapy. No special family history was reported. In admission to the Coronary Care Unit, she was hemodynamically stable and without signs of heart failure. Her blood pressure was 120/80 mmHg and her heart rate was 98 bpm.

**Figure 2:**
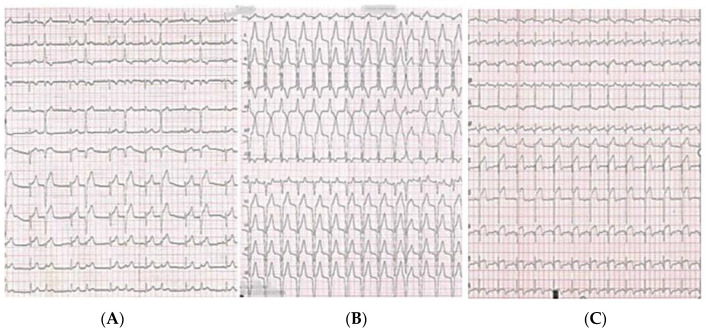
(**A**–**C**) The electrocardiogram (ECG) demonstrated sinus rhythm with ST segment elevation in anterior leads and premature ventricular beats (PVB) (**A**). The decision of the interventional cardiologist was not to perform any intervention. Slow ventricular tachycardia after coronary angiography was a sign of successful spontaneous reperfusion (**B**). On the fourth day of hospitalization, after mild physical activity, the patient felt chest pain. On ECG (**C**), ST elevation in the anterior segments developed (compared with the previous ECG performed at rest). Her blood pressure was 130/70 mmHg and her heart rate was 82 beats per minute. She was transferred to the catheterization laboratory again.

**Figure 3:**
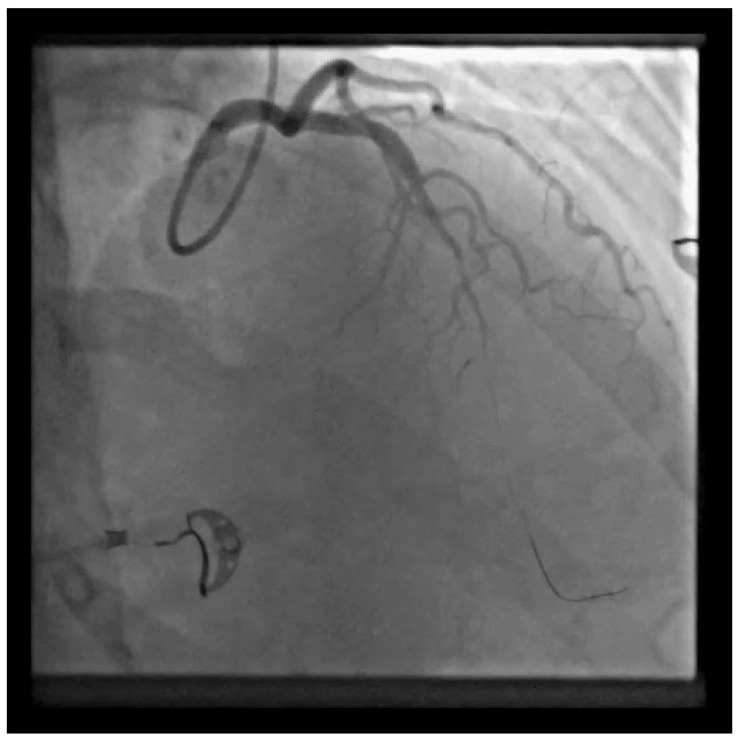
LAD SCAD from the medial to distal segment was observed along with lumen occlusion of the coronary artery; it was SCAD type 4. After the guide insertion, LAD was wired and dilated with balloon inflation (1.5 × 15 on 4 atm) (Video S2).

**Figure 4:**
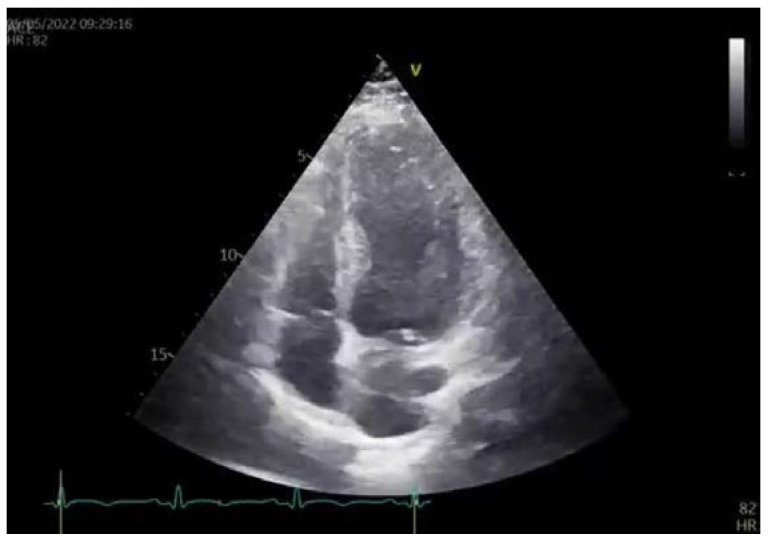
Transthoracic 2D echocardiography showed akinesia of the apical segments of all walls and there was akinesia in practically one-half of the left ventricle (LV). However, the thicknesses of akinetic walls were preserved. Left ventricle ejection fraction (LVEF) was 39%. The echocardiography exam is presented in Videos S3 and S4. Global longitudinal strain (GLS) was −9.7%. Apical segments of LV, assessed by 2D strain echocardiography, were blue-colored in the bull’s eye plot, which was a sign that these segments had completely impaired strain movements, while medial segments of LV had reduced strain movements. Basal segments of LV had good strain movement. Several routine blood and biochemical tests were conducted which showed the following values of two peaks of cardio-specific enzymes on the second and fifth days. Hormonal thyroid status was normal.

**Figure 5:**
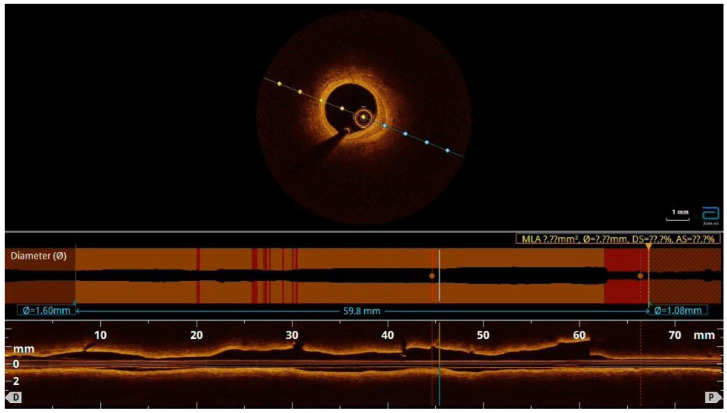
One mount later, the patient felt weak and tired and had atypical symptoms of angina. She had intermittent episodes of supraventricular tachycardia and NYHA II-III heart failure. She was admitted to the hospital and coronary angiography and optical coherent tomography (OCT) were performed. There was one partially absorbed hematoma without compression of the lumen in the LAD medial segment and without signs of persisted dissection (Video S5).

**Figure 6:**
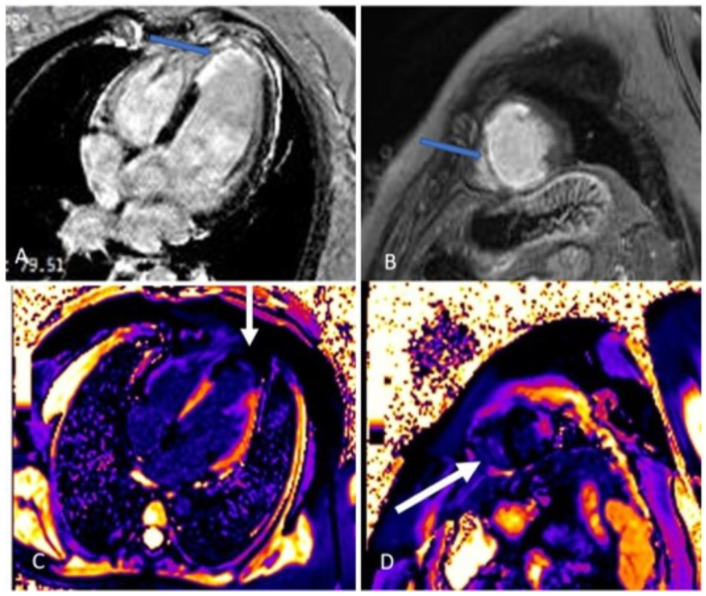
(**A**–**D**). The kinetic estimation by cardiac magnetic resonance (CMR) showed that apical segments of LV were akinetic to dyskinetic, and LVEF was 44% (Video S6). In figure parts (**A**,**B**), four chamber and short axis views are presented. Late gadolinium enhancement (LGE) was seen in the sub-endocardium in the medio-apical part of the septum and transmural LGE was seen in the apical parts of the septum and inferior, posterior, and anterior walls of LV (blue arrow). In figure parts (**C**,**D**), the zone of fibrosis, which was the zone of infarction, was present in apical segments of LV when using post-contrast T1 mapping (white arrow). Besides that, a high T1 signal was present in the peri-infarct area. Qualitative differences in infarct size which are calculated as (LGE mass/total LV mass) × 100 by CMR were discovered in the pattern of myocardial injury. In our patient, the size of fibrosis (infarct size) was 13%.

**Figure 7:**
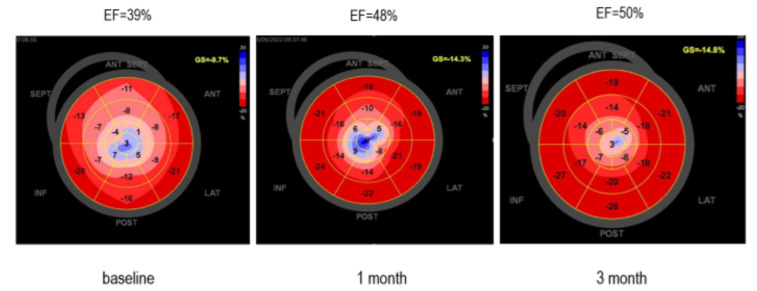
During the follow-up, echocardiography exams were performed and the progression of LVEF values from 38 to 50% as well as GLS from −9.7% to −14.8% was observed. The blue-colored zones in the bull’s eye plot estimated by strain were improved. The type of SCAD and TIMI flow grade may be correlated with myocardial infarct size [4]. About 26% of SCAD patients have a very small reduction in LVEF < 50% and over 50% have subsequent normalization of wall motion abnormality and LVEF on follow-up assessment [4]. Different pathophysiology of the SCAD ST segment elevation of myocardial infarction (STEMI) compared with Type 1 STEMI might be an explanation of the differences in infarct size and in infarct zone appearance. In SCAD, the forming of infarct size could be a balanced process. OCT studies showed that the absence of a fenestration in the vessel wall leads to increased false lumen pressure and compression of the true lumen, resulting in larger infarct size [5]. A hematoma which can compromise the true lumen without obstruction is often found. Furthermore, the absence of a conditioning effect and collateralization induced by a prior-fixed stenosis (as found in Type 1 STEMI) might tend to increase infarct size in the event of a SCAD-STEMI [6]. SCAD survivors with apparent endocardial sparing differ from STEMI patients with a typical pattern of endocardial involvement [6].

Inappropriate immune response to AMI scar formation could be the explanation for future LV remodeling and the infarct scar size. Sustained inflammation after AMI leads to LV remodeling and progressive cardiac dysfunction. Increased proinflammatory cytokines such as tumor necrosis factor (TNF)-α, interleukins (IL)-1β, IL-6, and monocyte chemoattractant protein-1 are strongly related to inappropriate healing of the myocardium after AMI. In patients with heart failure (HF), expression of TNF-α and tumor necrosis factor receptor (TNFR1) on activated CD4+ T cells is increased [7]. In post-AMI patients developing LV remodeling and eventually HF, two different types of cardiac CD4+ T-cells transmigration were observed. In the first three days, post-AMI CD4+ T-cells responded rapidly and returned to baseline by day 14, but in the case of HF, the second phase of their activation occurred, leading to further damage of cardiomyocytes. [8] The acknowledgment of the extensive immune response to myocardial damage after AMI leads to novel therapeutic targets, namely in the form of limitation of the cytotoxic CD8+ and heart-specific CD4+ T infiltration as well as targeted blockade of various cytokines (IL-1, IL-11, IL-15, TGFβ, etc.) or administration of reparative ones (IL-10) [9]. 

Myocardial strain is one of the principles for quantification of LV function, which is feasible with speckle-tracking echocardiography [10,11]. The GLS as a measure of systolic function may be a more useful and sensitive parameter than LVEF to identify sub-clinical LV dysfunction [11]. Strain recovery is impaired in the zone affected by infarction, as well as in parts of intramyocardial hemorrhage or microvascular obstruction. In our case, the values were improved, but this did not lead to a return to normal.

The management of SCAD has been a paradigm shift during the previous decade [12]. The reason for that may be unpredictable clinical courses and outcomes. The challenge is to find predictors of the risk of SCAD recurrence, as well as to find predictors of larger infarct scars. Patients with recurrent clinical symptoms of chest pain, ST elevation, and complications represent a special group of interest. Special consideration of possible treatment of this subgroup of patients has to be taken. The decision to revascularize patients with SCAD is challenging, given the associated complications and technical difficulties [13]. Therapeutic approaches for SCAD range from the ”watch and wait” method to complete revascularization with implantation of one or more stents or aortocoronary bypass grafting. In a previous study, 53% of SCAD patients underwent PCI and 7% CABG [14]. Patients treated with dual antiplatelet therapy had a significantly higher risk for future major adverse coronary events compared with those treated with single antiplatelet therapy during one year follow-up [15]. 

To conclude, SCAD represents a significant cause of AMI with a challenging diagnostic and therapeutic approach. Multimodal imaging for the establishment of infarct size and its development is crucial.

## Data Availability

Not applicable.

## Supplementary Materials

The following supporting information can be downloaded at: https://www.mdpi.com/article/10.3390/diagnostics13091518/s1. Video S1: First coronary angiography. Video S2: Second coronary angiography. Video S3 and S4: Baseline echocardiography exam. Video S5: Coronary angiography with optical coherent tomography (OCT). Video S6: Assessment of left ventricle function by cardiac magnetic resonance.
